# Supplementary material for: Are carbon emissions trading and green financial instruments synergistic? -Comprehensive quantitative research based on content analysis
Source: PLoS One. 2024 Mar 7;19(3):e0298601. doi: 10.1371/journal.pone.0298601 (PMC10919601; doi:10.1371/journal.pone.0298601)
Supplement: S1 Data — (ZIP) [file pone.0298601.s001.zip › Data/地级市Z_F工作报告.zip!/╜¡╦╒/─╧═¿╩╨/└┤╫╘╣1⁄2╓┌║┼í╛┬φ┐╦╩2╛▌═°í┐_2004.docx]

各位代表：

　　现在，我代表市人民政府向大会作工作报告，请予审议，并请市政协各位委员和其他列席人员提出意见。

　　一、2003年工作回顾

　　过去的一年是不寻常的一年，是我市国民经济在多年蓄势的基础上强劲增长的一年，也是南通争当全省江北"两个率先"排头兵取得显着成效的一年。在省委、省政府和市委的正确领导下，全市上下高举邓小平理论伟大旗帜，全面贯彻"三个代表"重要思想，深入贯彻党的十六大精神，按照"依托江海、崛起苏中、融入苏南、接轨上海、走向世界、全面小康"的总体思路，围绕市委确定的方针、重点和市十二届人大一次会议提出的阶段性任务，面对非典疫情的严峻考验和复杂多变的经济形势，解放思想，抢抓机遇，奋勇争先，攻坚克难，全面超额完成年度目标计划，全市实现GDP超千亿、财政收入超百亿的历史性突破，经济社会进入跨越发展的起飞阶段。

　　（一）国民经济跃上新平台，多项主要指标超预期、超历史。国民经济总量规模及结构状况发生具有历史意义的阶段性变化。全市完成GDP1006.65亿元，比上年增长13.4%，增幅为7年来之最，比上年提高2.3个百分点。一二三产业增加值分别增长4.1%、16.8%和12.6%，第二产业占比超过50%，第一产业占比首次调整到15%以内；实现财政收入116.99亿元，增长21.7%，占GDP比重首次达到两位数，为11.6%。农业结构调整和产业化进程加快，项目农业强势推进，龙头企业迅速壮大，园区建设加速发展，农业产业链拓展延伸，传统农业正加快向现代农业迈进。实施以工兴市第一方略开局良好。全年完成工业增加值452.37亿元，增长17.7%，增幅为7年来之最，在全省的位次前移5位；规模以上工业企业实现利润增长53.4%，经济效益综合指数达到146.2%，为历史最高水平。建筑业市场开拓能力增强，全年完成施工产值450亿元，增长15.7%。服务业实现较快增长，增幅高于全省水平0.9个百分点。商贸流通业升温加速，旅游会展业进一步活跃，房地产业持续健康发展。全市社会消费品零售总额达331.28亿元，增长12.7%，增幅为6年来之最。县（市）域经济发展全面加速，工业增加值在全市占比达69.7%，提高0.3个百分点。

　　（二）固定资产投资强势突破，工业投入增幅列全省前列。继续坚持猛攻投入，全市完成全社会固定资产投资448.42亿元，增长44.7%，增幅为7年来之最，其中规模以上投资达318.71亿元，增长71.8%。工业投入出现了多年未有的强劲势头，全年完成工业投入240.37亿元，增长94.2%，增幅居全省前列，占全社会固定资产投资的比重从长期以来的30%左右提升到53.6%。农业投入也出现了多年未有的好势头，工商资本、民间资本和外商资本投入农业总额增长90%。各级各类开发区和乡镇工业园区基础设施投入增长62.8%。金融等部门全面扩大信贷投放，年末贷款余额达726.87亿元，增长38%。

　　（三）市场化改革向纵深推进，民营经济发展全面提速。继续强攻各项改革，全力增强体制创新第一动力。大力度推进国有企业改革，国有资本退出一般竞争性领域和劣势企业退出市场目标基本实现，"大而盈、大而亏、大而难"企业改革取得重大突破，跨地区、跨行业、跨所有制资产重组步伐加快，150多家市属国有企业完成深化改革任务，退出国有资本近10亿元，置换职工身份3.3万人。事业单位改革走在全省前列，108家市属生产经营型事业单位改制转企任务全部完成；公益型事业单位改革正在加快推进。现代市场体系建设、规范和整顿市场经济秩序取得新成效。鲜明提出争创江苏民营经济第一大市的奋斗目标，各项工作全面推进，民营经济大发展的浓烈氛围加速形成，全市私营企业数、注册资金额分别增长23%、74.9%，累计分别达26962家、279亿元。民营经济增加值在GDP中的占比增加3.5个百分点，达到37.1%。

　　（四）利用外资实现"撑杆跳"，开放型经济正在重返全省第一方阵。强势推进招商引资，利用外资创历史新高。全年新批注册外资突破23亿美元，注册外资实际到帐额7.31亿美元，分别增长367.4%和206.5%，增幅均居全省第一。新批总投资超千万美元的外资大项目98个，是上年的3倍多。总投资18亿美元的王子制纸项目成功落户我市，这是改革开放以来我市最大的利用外资项目，也是迄今日本对华投资的最大项目。对外贸易保持较快增长，进出口总额达51.64亿美元，增长32.6%，其中出口32.85亿美元，增长29.8%。外经工作各项指标继续位列全省之首，新签对外承包工程和劳务合同额、营业额分别增长34.6%和39.5%。海关、国检、海事、边检等口岸联检单位整体联动，大通关建设取得新成效。外事、侨务、对台事务等工作在扩大开放中发挥了积极作用。

　　（五）新一轮江海开发和接轨上海全面启动，一批重大项目取得实质性进展。呼应全省沿江开发战略，全力抢抓第一机遇，加快沿江开发、江海联动，高起点制定《南通市沿江开发详细规划》和产业、交通、港口等专项配套规划，扎实推进7个方面48项重点工作，岸线整合整治开发工作全面推开，岸线资源管理得到加强。加速港口建设，狼山港区三期工程等8个泊位建设进展顺利，南通港货物吞吐量突破5000万吨，增长33.7%。洋口港、吕四港开发建设取得实质性进展。沿江沿海产业集聚进程加快，新批总投资超千万美元外资项目有90%以上落户在沿江沿海地区。重点实施的37个重大项目进展显着，其中30个项目相继开工建设，如东LNG（液化天然气）、启东大唐电厂等特大型项目已进入最后审批阶段。海洋经济和生态农业得到新的发展。跨江合作、区域联动取得新进展，南通、苏州互动合作开发协议正式签订并开始实施。实施《南通市接轨上海工作纲要》初见成效，农业、科技、人才、交通、园区、港口、旅游等方面接轨步伐加快。

　　（六）基础设施建设快速推进，城市建设投入创历年之最。突出城市第一载体建设，调整城市总体规划，中心城市建设全面升温，呈现老城区、新城区、开发区、狼山风景区、港闸区、观音山区域竞相推进的良好局面。市区城建投入超过100亿元，其中政府主导性投资接近40亿元，超过前5年投入总和。经营城市迈出新步伐，市区首期城建项目招商协议引进外资、民资折合人民币17.25亿元；土地招商创下总收益、土地单价等5项历史记录。大力实施城市景观工程、洁净工程，开展环境集中整治，市容市貌进一步改观。加强生态环境建设，国家环保模范城市创建工作开始起步。市区建成区新增绿地120公顷，绿化覆盖率达37%；大力实施城区河道整治，水环境进一步改善。各县（市）县城和中心镇建设力度加大，步伐加快。全市城市化率达42%，提高近3个百分点。重点基础设施建设加快推进。全市交通基础设施建设完成投资30.28亿元，为历年之最。苏通大桥主桥及北接线工程全面开工，崇启、崇海大桥前期工作取得进展，通常汽渡迁址工程超常推进，皋张汽渡前期工作全面启动；通启、盐通高速公路加快建设，海防公路全线贯通，沿江公路提前开工；新长铁路正式投入货运，宁启铁路一期工程铺轨结束。积极争创"畅通工程"优秀管理等级城市，道路交通环境不断改善。加强土地管理，土地资源得到集约利用和有效保护。

　　（七）防治非典取得重大胜利，应急处置能力显着增强。面对突如其来的非典疫情，在市委的领导下，坚决贯彻中央、省一系列重要决策和部署，沉着应对，全力以赴，紧紧依靠全市各级组织和广大人民群众，立足于"早、快、严、实"，建立了统一高效的组织指挥体系、反应敏捷的应急救治体系和盖边沉底的群防群控体系，牢牢把握防治工作主动权，实现了"非典疫情不扩散、非典患者零死亡、医护人员零感染"的目标，保持了安定团结的政治局面和良好的经济社会秩序。同时，立足于建立长效防治机制和更好地应对突发事件，制定并落实《南通市公共卫生突发事件应急预案》及其他方面的预防性措施，提高了政府处置重大公共突发事件能力。尤其可贵的是，在抗击非典斗争中全市上下形成了"万众一心，众志成城，团结互助，和衷共济，迎难而上，敢于胜利"的抗非精神，激励我们在前进的征程中不畏艰难，夺取经济建设和社会发展的新胜利。

　　（八）各项社会事业全面发展，精神文明和民主法制建设得到加强。继续大力实施科教兴市战略，精心组织科技项目实施，204个项目被列入省级以上科技计划；加快技术创新体系建设，大力开展产学研活动，加快高新技术产业发展和新技术推广运用，科技合作项目比上年翻一番，科技进步对农业、工业的贡献率分别达54%和44%，市及各县（市）区均通过全国科技进步考核。社会科学研究取得新进展。基础教育办学水平继续提高，"以县为主"的农村义务教育管理体制得到较好落实，初中毕业生升学率达82.9%，全市高考主要指标稳定在全省第一位置；职业教育、成人教育稳步发展，终身教育体系加快构建；南通大学园区一期工程开工建设，综合性大学申报工作顺利推进，引进和培养高层次人才取得显着成绩。文化大市建设成效明显，环濠河文博馆群等重点文化设施建设稳步推进，广播电视产业化迈出新的步伐，文艺、新闻精品生产在全省保持领先。省十六运承办工作开始启动，竞技体育成果丰硕，全民健身服务体系向乡镇延伸。预防保健水平初步达到基本现代化标准，新型农村卫生服务体系不断完善。人口与计划生育工作取得新成绩，在全国率先建立计划生育特困家庭救助机制，计划生育率达99.2%。民族、宗教、统计、审计、防震减灾、无线电管理、气象、档案、保密、地方志等工作进一步加强。以公民道德建设为核心，全力打造全省江北第一文明城市群，新时期精神文明建设"南通现象"得到进一步弘扬。国防教育、人民防空、征兵、民兵预备役和优抚安置工作不断加强，再获全国"双拥模范城"称号。坚持依法治市、依法行政。自觉接受人大及其常委会的法律监督、工作监督和政协的民主监督，认真执行人大及其常委会决议，切实抓好人大议案和人大代表建议、批评、意见以及政协委员提案的办理工作，市级办理满意和基本满意率分别达95.3%、94.4%。民主党派、工商联和无党派人士参政议政的作用得到充分发挥，与工会、共青团、妇联、科协等群众团体的联系日益密切。开通"市长电子信箱"，向社会公开征集对政府工作的建议，大力推进政务公开。高度重视并认真解决群众关心的热点、难点问题，强化信访工作责任制，积极妥善处理群访、集访和越级上访事件。安全生产进一步加强。着力构筑社会治安大防控格局，首创社会矛盾纠纷大调解机制，创建"平安南通"和全省最安全城市成效显着，万人发案率全省最低，第六次获得全省社会治安综合治理先进市称号。

　　（九）就业和社会保障工作扎实推进，人民生活水平进一步提高。坚持把就业作为民生之本，制定并落实促进就业再就业的政策措施，保持了就业形势的基本稳定。全市城镇共提供就业岗位12.55万个，有4.46万人实现就业再就业，再就业率达63.3%，年末城镇登记失业率为3.75%。农村劳动力转移净增10.76万人，累计达181.87万人，占农村劳动力总数的62.3%。社会保障体系不断完善，保障能力继续提升，"两个确保"进一步巩固。城乡居民收入较快增长，市区居民人均可支配收入9598元，增长11.1%；农民人均纯收入4393元，增长6.3%，是近5年来增长最快的一年。年末居民储蓄存款余额达882.12亿元，人均11325元。城乡市场繁荣，居民消费水平提高，人民生活质量明显改善。切实保障弱势群体基本生活，健全社会救助体系，城乡最低生活保障制度、城市廉租房制度等政策得到落实。农村五件实事扎实推进，完成农村草危房改造6987户、16640间；农业特产税改征农业税、农业税社会减免先减后征、户户减负、村组三项资金管理等政策已全部落实到位；新型农村合作医疗试点工作成效明显，试点县覆盖率达87%；新增农村改水受益人口56.81万人；农村公路竣工通车里程536.52公里，完成省下达年度目标的111.2%。妇女、儿童、老龄、残疾人、关心下一代、红十字和慈善工作取得新成绩。

　　（十）政府职能加快转变，综合发展环境日趋优化。根据市委关于争创全省最佳办事环境的要求，以"创新、规范、高效、廉洁"为目标，注意加强自身建设，致力于建设服务型政府，强化机关作风建设，办事环境不断优化，便民利民措施正继续得到落实，"重商、亲商、安商、扶商"的氛围加速形成。行政审批制度改革继续深化，以全省最优为标准进一步清理审批事项，推行告知承诺制、并联审批制，审批速度在上年基础上加快三分之一。市级机关部门和行政管理型事业单位的"清实体、清资产、清资金"工作扎实推进。强化狠抓落实第一基本功，加强调查研究，完善重点工作、重大项目的推进、督查、考评机制，有效保证了各项关键举措的落实。认真落实党风廉政建设责任制，加大反腐倡廉力度，进一步树立了政府良好形象。按照市委决策部署和市十二届人大一次会议议案要求，精心打造"中国近代第一城"崭新形象，全面展开内涵挖掘、宣传推介、建设保护工作，通过多种形式搭建平台，成功举办2003中国南通港口经济洽谈会和纪念张謇先生诞辰150周年等重大系列活动，邀请国际国内知名人士和重要客商亲临南通，切身体验南通悠久的人文历史和崭新的城市面貌，并与国家级重要媒体联手开展高起点、高规格、高频率的宣传系列活动，向世界强势推介南通。"中国近代第一城"城市品牌效应和要素集聚效应凸现，南通的城市影响力迅速提升，南通在国内外的知名度和美誉度大大提高。

　　各位代表，过去一年所取得的成绩，是在省委、省政府和市委的正确领导下，在市人大、市政协的监督支持下，在历届政府工作的基础上，全市上下励精图治、艰苦奋斗的结果，凝聚着广大创业者、劳动者、建设者的心血和汗水，展示了南通人民勇于创新、开拓进取的时代风采。在此，我谨代表市人民政府向辛勤工作在各行各业的广大干部群众，向给予政府工作大力支持的市人大代表、市政协委员和离退休老同志，向关心、支持南通现代化建设的部、省驻通单位和解放军指战员、武警官兵、公安民警及社会各界人士，向积极参与南通发展的港澳台同胞、海外侨胞和国际友人，表示崇高的敬意和衷心的感谢！

　　各位代表，在充分肯定成绩的同时，我们也清醒地看到，我市经济社会发展中还存在不少矛盾和问题，政府工作中存在的不足有待于加快改进。与先进地区相比，我市经济增长速度还不够快，人均指标差距仍然较大，经济发展后劲有待进一步增强；中心城市、中心城镇的集聚和辐射带动作用还不够强，产业发展的承载能力有待加强；社会事业发展的活力还不够，农村教育、文化、卫生等事业发展不够平衡；就业和社会保障的压力仍然较大，城乡居民收入与全面小康的要求还存在较大差距，特别是农民增收仍比较困难；政府职能转变和机关作风建设还不能适应改革和发展的要求，市级机关办事"中梗阻"现象时有发生，行政效能和服务水平有待进一步提高。对此，我们将高度重视，采取扎实措施，认真加以解决。

　　二、2004年主要任务

　　今年是全面贯彻党的十六届三中全会精神的第一年，是实施"十五"计划的关键一年，也是南通经济社会跨越发展的重要一年。今年政府工作的总体要求是：以邓小平理论和"三个代表"重要思想为指导，全面贯彻落实党的十六大、十六届三中全会、中央经济工作会议和省委十届六次全会精神，按照市委确定的总体思路、奋斗目标、工作举措和全市经济工作会议的具体部署，继续坚持猛攻投入、强攻改革、扩大开放、优化环境的方针，进一步解放思想，开拓创新，争当全省江北"两个率先"排头兵，积极推动市场化、工业化、国际化、城市化和农业产业化进程，加快发展步伐，提高增长质量，不断改善城乡人民生活，促进经济社会全面、协调、可持续发展，努力开创南通跨越发展的新局面。

　　今年全市国民经济和社会发展的主要预期调控目标为：地区生产总值增长13%以上，财政收入增长15%以上，全社会固定资产投资增长30%以上，社会消费品零售总额增长13.5%以上，注册外资实际到帐额力争翻一番，城镇居民人均可支配收入增长11%，农民人均纯收入增长8%，城镇登记失业率控制在4%以内。

　　做好今年的各项工作，实现经济社会跨越发展的阶段性目标，必须注意把握好以下几点：

　　--必须增强抢抓机遇加快发展的历史责任感。胡锦涛同志指出，历史一再表明，机遇极为宝贵，稍纵即逝。在历史发展的关键时期，把握住了机遇，就有可能实现跨越式发展；而丧失了机遇，就会成为时代发展的落伍者。面对南通跨越发展的历史机遇，我们必须十分珍惜，树立更加强烈的责任意识和机遇意识，只争朝夕，坚定不移推进跨越发展，实现南通在全省乃至长三角板块的争先进位。

　　--必须围绕跨越发展凝心聚力。抢抓跨越发展的历史机遇，实现"两个率先"的目标任务，必须把全市人民的智慧力量凝聚到跨越发展上，把各种资源和要素汇聚到跨越发展的进程中。以贯彻落实党的十六大和十六届三中全会精神为推动，开辟解放思想的新空间，满怀热情地支持探索，鼓励创新，善待挫折，确立新的人才观，发挥好各方面加快发展的积极性，引导和保护好全市上下高涨的发展热情，千方百计满足人民群众强烈的致富愿望，继续保持奋勇争先的精神状态和昂扬向上的旺盛斗志，鼓实劲、出实招、求实效。

　　--必须坚持全面、协调、可持续发展。在推进跨越发展的过程中，要坚持新的科学发展观，坚持以人为本、富民为先、执政为民，把中央提出的"五个统筹"和省委、省政府提出的"四个协调"的要求贯穿始终，正确处理好经济增长与加快富民的关系，正确处理好经济发展与社会发展的关系，正确处理好加快发展与提升质态的关系，正确处理好改革发展稳定的关系，在加快发展中给人民群众带来更多实惠，在深化改革中解决好新的矛盾，让人民群众在更多的方面分享到经济增长的成果。毫不动摇地继续猛攻投入、强攻改革、扩大开放、优化环境，一着不让地抓好增量增长和潜力增长，夯实发展基础，构筑发展优势，激发发展活力，为实现跨越发展提供更加坚实的支撑。

　　围绕跨越发展的要求和今年目标任务的实现，重点抓好以下工作：

　　（一）以猛攻投入为重点，推进工业经济再提速。大力实施以工兴市第一方略，坚持在发展中推动结构调整升级，加速工业化进程，全市规模以上工业增加值增长18%、力争达到19%，工业在地区生产总值中的占比提高1.2－1.5个百分点。一是继续毫不动摇地推进总量扩张。加快培育一批新的经济增长点，力争全年规模以上企业净增300家以上，新增年销售收入超亿元企业40家以上，超10亿元企业达到10家。进一步提高工业经济的组织程度，努力解决电力、能源、用地、资金、运输等要素瓶颈制约，促进工业提速增效。二是注重放大优势提升增长质态。突出做强产业集群和板块经济，重点培育能充分发挥比较优势的临港型基础产业、制造业和已形成的电子产业板块、生物医药板块。坚持走新型工业化道路，强化科技的支撑作用，组织实施科技企业孵化器建设工程、高新技术产业发展"456"工程、产学研合作"121"工程、制造业信息化示范工程，推动传统产业高新化和高新技术产业化进程。加强企业家队伍建设，培育名人、名牌、名企，促进企业做大做强。三是坚持不断扩大投入产出绩效。全市工业投入350亿元以上，工业投入占全社会固定资产总投资的60%以上，规模以上工业投入增长60%以上，技术装备投入占工业投入的60%以上。重点推进产业突破型项目、重大国债项目、投资超千万美元项目和单位投资密度大的项目。注重优化投向，提高投效，尽可能缩短投入产出周期、提高投入产出比、增强投入即期效应。全力推进50个投资超亿元项目，促其尽快投产；重点跟踪100个投资超2000万元工业项目，实现100%开工、50%竣工。

　　充分发挥南通建筑"铁军"品牌优势，着力培育一批资金实力雄厚、专业配套合理、总承包能力强的大型企业及企业集团，力争一级企业新增3－4家、特级企业有所增加，在拓展经营领域和延伸产业链上加快突破，提高施工总承包能力和专业施工承包能力，尽快由"建筑大市"走向"建筑强市"。

　　（二）以突破体制机制瓶颈为重点，推进改革再深化。按照完善社会主义市场经济体制的要求，继续保持和增创体制改革的先发优势，更大程度地解放生产力。一是全面完成国有企业改革任务。加快"大而盈、大而亏、大而难"企业改革进程，确保上半年改制到位。坚持阳光操作，以增量式、开放式改革吸引外资、民资参与国企改革，促进企业投资主体多元化，真正建立现代产权制度，保证国有企业改革健康、有序、规范进行。全面整合国有资产营运机构，实现国有资产管理全覆盖，建立新型国有资产营运体系。二是加速推进社会事业改革。按照社会化、多元化、市场化改革方向，加快实施公益型事业单位分类改革，保持事业单位改革在全省的领先地位。深化科技、教育、文化、卫生、广电等领域改革，鼓励社会资本参与事业单位改革，不断增强社会事业发展活力。三是大力推进财政管理体制和投融资体制改革。按照事权、财权一致的原则，积极推进市区财政体制改革。继续推进和完善部门预算、政府采购等举措，清理行政收费，建立健全公共财政体制。规范政府投资行为，强化财政投入的绩效评估。打破所有制界限，全面开放经营性基础设施和社会公用事业建设、营运市场。在产业投入和城市建设等方面尽快形成政府引导、社会参与、市场运作的投资体制。推动企业通过多种形式直接融资，力争1－2家企业股票发行上市。四是全力创新行政管理体制。按照"经济调节、市场监管、社会管理、公共服务"四大职能，进一步解决政府职能越位、缺位和错位的问题，做到"有所为，有所不为"。加快完善城市管理综合执法体制，积极推进农业管理领域综合执法试点。

　　（三）以招商引资为重点，推进开放型经济再跨越。适应入世以后经济国际化进程加快的形势，进一步扩大开放，加快南通走向世界步伐，加速重返江苏开放型经济第一方阵进程。一是全力打好新一轮利用外资攻坚战。继续保持高位推动招商引资的良好态势，再掀招商引资新高潮。精心组织首届世界大城市带高层论坛和2004中国南通港口经济洽谈会，更有针对性地举办好赴日、韩和港、台以及欧、美地区的系列招商活动，努力实现重大引资项目的新突破，力争新批超千万美元项目100个，其中超亿美元项目3－5个。在强势推进招商的同时，继续为现有外资企业提供优质高效的服务，鼓励其增资扩股，不断拓展发展领域，打开"以外引外"新局面。积极发挥外事、侨台作用，吸引更多的境外客商来通投资。二是大力推动外贸出口和外经工作跨上新台阶。加快出口产品结构调整，最大限度地降低出口退税机制调整对我市出口的影响，实现外贸出口增长15%以上。加快大通关建设，提升口岸服务功能，优化出口环境。推动科技型等劳务输出新突破，在巩固传统劳务市场的基础上，努力开拓新市场，大力发展境外加工和贸易企业，全力推动外经总量扩大、质量提升，保持外经在全省的领先优势。三是着力加快接轨上海步伐。全面实施《南通市接轨上海工作纲要》，积极参与长三角经济一体化分工，加速推进与上海在政策、产业、要素和交通等方面的对接，在人才、信息、旅游、技术产权交易等方面的资源共享。超前谋划过江通道建成后承接上海产业梯度转移和传递辐射的举措。继续大力实施通启高速、盐通高速南通段、沿江公路等高等级公路及干线网化工程建设，完成通常汽渡迁建，实现皋张汽渡通渡，推进苏通大桥按期顺利建设，积极推进崇启、崇海大桥开工建设的前期工作，开工建设宁启铁路南通至南通东及狼山港铁路支线工程，积极开展沪通铁路前期准备工作，加快缩短与上海的时空距离。

　　（四）以全民创业为重点，推进民营经济发展再进位。按照省委、省政府的"六放"要求，围绕争创江苏民营经济第一大市的目标，学习浙江经验，推动"思想彻底解放、政策彻底放宽、服务彻底到位"，实现民营经济发展"一个提升、一个领先、一个前移"，即民营经济增加值在地区生产总值中的比重提升到40%以上，民营经济增长速度在全省领先，民营经济总量在全省的位次前移。一是推动全民创业，构筑"铺天盖地"的总量优势。在全社会营造"崇尚财富、创业至上"的浓厚氛围，激发人民群众的创业热情。开展民营经济"先进县（市）区、标兵乡镇、明星村、百强企业、优秀企业家和最佳服务部门"评比竞赛活动。全面落实《关于争创江苏民营经济第一大市的若干意见》，降低市场准入门槛，营造公平竞争环境，解决融资难、用地难等方面的突出矛盾，提供支持创业、便利创业的各类服务，最广泛地吸纳市外资本，最充分地激活本地民资，新增个体工商户3万户、私营企业8000家、注册资金120亿元。二是着力扶强做大，构筑"顶天立地"的规模优势。积极引导民营企业以多种方式广泛集聚货币资本、实物资本、技术资本、人力资本，增强核心竞争力，扶持培育一批"规模型、科技型、外向型"的骨干民营企业群体，使之成为全省乃至全国行业"单打冠军"。三是加快培育区域特色，构筑产业集群优势。突出特色，壮大规模，鼓励民营企业延伸产业链，提升产业优势，进一步拓展床上用品、纺织服装、五金电动工具、花木盆景、海洋产品深加工等特色产业集群，形成区域特色块状经济。四是建设大型市场，构筑市场带动优势。充分发挥区位优势，加快各类专业市场建设，用专业市场带动一片产业、活跃一方经济。加快完善融资担保、信息咨询、人才培训、技术支持等社会化服务体系，加快建立信用征信系统，为民间资本孵化和民营经济发展提供支撑。

　　（五）以主攻大项目为重点，推进江海开发再突破。紧紧抓住沿江开发第一机遇，全面实施《南通市沿江开发详细规划》，实现沿江前沿地区经济总量、岸线和园区投资强度、开发区产业集中度和港口吞吐量"四个提升"，确保沿江前沿地区生产总值增长17%以上；固定资产投资增长40%以上，其中工业投入增长80%以上；实际利用外资增长一倍以上；港口货物吞吐量达到6000万吨，增长20%，集装箱货运量有较快增长。一是统筹协调开发、整治、整合、管理工作，不断提高集约化开发水平。江海岸线资源是滨水城市的真金白银，必须十分珍惜地开发好、利用好、管理好，明确投资强度要求和刚性控制标准，加强对岸线开发、使用的规范和引导。加快狼山港三期主体工程建设，快速推进如皋港、洋口港、吕四港等江海港口的开发。加快新通海沙和新开沙夹槽整治开发基础工作，加速形成新的可利用岸线。认真落实《关于进一步加强江海岸线管理的试行意见》，加快建立岸线、码头回收整合机制，制订泊位建设标准，确定港口岸线等级及有偿使用办法，提高集约开发水平。治理和保护生态环境，确保江海资源可持续开发。加快大型排海设施的论证实施，扩建市区污水处理厂，加快排污管网系统建设，保护长江取水口生态环境。二是突破重大项目，促进产业结构优化。沿江着力抓好王子制纸、华能三期、醋纤四期、永兴多媒体扩建等一批项目，沿海着力抓好如东LNG、风力发电一、二期和启东大唐电厂等项目，以产业主导性强、带动作用大的项目的突破，推进江海开发。三是整合各类园区，提高产业集中度。强化产业规划引导，着力抓好国家级、省级开发区和乡镇工业园区的规划建设，合理划分产业发展方向，加快形成规模效应和产业联动效应。围绕提升集约开发水平，完善相关政策措施，形成利益共享机制，鼓励和引导项目向功能园区集聚，有针对性地对现有工业园区适当进行合并重组。加快园区基础设施建设步伐，构筑平台，集群发展，着力提高开发区和工业园区项目的投资强度和产出水平。四是完善合作机制，加快区域联动开发。健全南通、苏州两市互动合作联席会议制度，推动《南通、苏州两市沿江开发互动合作框架协议》的落实，着力加快过江通道节点的对接和产业园区的跨江联动发展。按照两地市、县（市）之间的区域特色和产业特点，在招商引资、项目引进和港口基础设施建设上加强协调，优化工作机制。

　　（六）以市区和县（市）城建设为重点，推进城市形象再提升。统筹城乡发展，优化城市体系布局，加快城市化进程，全力打造"中国近代第一城"崭新形象，城市化率达到45%以上。一是进一步完善城市规划。按照建设百万人口大城市的目标，充分发挥规划在城市化和城市现代化进程中的指导和调控作用。适应沿江开发和城市化发展的要求，按照上海都市圈副中心城市的定位，完善城市的总体规划、分区规划和控制性详规。做好产业布局规划，通过有力措施和政策引导，促进人口向城市集中、产业向园区集中，确保要素集聚服从并服务于城市化进程和大中小城市体系格局的形成。高度重视、研究制定沿江、沿海、沿河滨水地区的规划布局，正确处理生产岸线与生活岸线、产业集聚与人口集群、产业布局与自然生态资源的关系，最大限度使城市三维空间与城市资源有效利用紧密结合。同时做好各县（市）区域发展和空间规划的指导工作，使之与全市总体发展要求相衔接。严格执行土地利用总体规划，坚持集约利用土地资源，在确保发展用地需要的同时，发挥土地资源的最大效益。二是加大城市建设力度。继续保持城建大投入态势，确保市区城建投入总量超百亿。加快中心城市建设，全力推进"五城（国家卫生城市、全国文明城市、国家环保模范城市、国家园林城市、国家历史文化名城）同创"。以体育会展中心和南通大学园区一期工程建设为重点，加快新区核心区开发建设。以"濠河十景"和滨江公园、山水间体育花园建设为重点，加快濠河、狼山风景区开发建设，创造精品特色。以打通城区之间连接线和整治"五横五纵"主干道为重点，加快老城区和港闸区改造出新，促进各区域和组团的融合。围绕各县（市）城建成30－50万人口的中等城市的目标，提高产业承载能力，努力形成中心城市、县（市）城、重点中心镇梯度发展、布局合理、功能互补的市域城镇体系，加快城市化进程。深化城镇户籍制度等方面的改革，逐步打破城乡二元结构，加快城乡一体化进程。三是提高城市经营管理水平。充分经营城市，构筑城建项目融资新平台。继续将公用事业、综合开发、冠名权转让、新区核心商务区四大类项目推向国内外招商，推动外资、民资和社会资本投入城市建设，引入竞争机制，增强发展活力。加强城市管理，大力推进社区建设，改善社区居委会工作和服务条件，使社区更快更好地承接社会保障、社区服务、社会管理功能。同时深入推进城市管理综合执法，进一步落实城市长效管理措施，着力整治交通秩序、市容秩序、新村出入口和集贸市场周边秩序，提高城市管理的效率和水平。四是大力促进城市繁荣。继续大力发展就业容量大的传统服务业、知识密集度高的新兴服务业、为制造业配套的生产服务业。大力发展物流仓储业，抓紧建设南通港港口综合物流园区等3大物流园区。整合壮大旅游业，全年接待海内外游客600万人次，旅游总收入50亿元。大力发展金融、保险、中介服务业，加快服务品种创新，加强银企协作，为金融业支持地方经济发展创造良好的诚信环境。积极发展以保洁绿化、家政服务、卫生保健、托老托幼、物业管理为重点的社区服务业。大力培育住房、汽车等消费热点，进一步扩大城乡居民的现代消费需求。确保服务业增加值增长13%以上，占地区生产总值比重提高0.5个百分点，服务业从业人员占全社会从业人员比重比上年提高2个百分点。

　　（七）认真做好"三农"工作，着力促进农民增收。按照全面建设小康社会的要求，全面发展农业和农村经济，继续把促进农民增收作为解决"三农"问题的中心任务抓好。一是进一步拓展农民增收的途径。以项目农业为抓手，充分挖掘地方农业资源，加快农业产业化进程，发展优质高效农业。加强农产品基地和主导、特色产业带建设，建立健全农产品质量安全体系。重点培育一批规模大、起点高、带动力强的农业龙头企业，力争20家销售收入超亿元。加速利用"三资"开发农业，建成一批亩均产出5000元以上的现代农业园区。发展壮大农民专业合作经济组织和农产品经纪人队伍，促进农产品市场流通。通过积极扶持农民自主创业和大力发展劳务输出，加快农村劳动力转移步伐。二是进一步提高农民增收的能力。切实加强农村基础教育和职业培训工作，提高农村人均受教育年限和劳动者技能素质，提升农村劳动力转移层次，更大程度上推动农村劳动力转移由体力型向专业型、技能型转变。加强农技队伍建设，完善农技推广服务体系。三是进一步改善农民增收的外部条件。增加对农业和农村的投入，提高农业机械化水平，加大县乡河道疏浚力度，加快改善农村基础设施条件，将新增社会事业投入向农村倾斜；加快推进农村五件实事，确保财政资金投入到位，让农民及早得益受惠；加大耕地保护力度，提高粮食综合生产能力；加强乡（镇）村基层组织建设，积极创造条件增加村级干部报酬；组织农村信用社改革试点，逐步将其改造成为农村服务的地方性金融企业。四是进一步保障农民的合法权益。巩固农村税费改革成果，探索实行市县财政补贴、乡（镇）村出资代交等办法，进一步减轻农民负担。完善土地承包制度，健全农村土地流转机制，加快推行被征地农民基本生活保障制度，切实维护失地农民的合法权益。加快推进沿海捕捞渔民转产转业工作。积极搞好扶贫工作和老区开发。

　　（八）加快发展社会事业，着力推动经济社会协调发展。按照"五个统筹"的要求，大力实施科教兴市、人才强市战略，加快社会事业发展步伐，建立与知识经济相适应的创新体系。一是着力构筑人才高地。尊重劳动、尊重知识、尊重人才、尊重创造，不断创新人才工作的观念、体制、机制和环境，紧紧抓住培养人才、引进人才、用好人才三个环节，建设好行政领导干部、企业经营管理者、专业技术人才三支队伍，强化高层次人才队伍建设。面向经济建设主战场，优化人才服务工作，建立和完善人才柔性流动机制，强化人才资源的市场化配置，做到人尽其才，才尽其用。二是加快建设科技创新体系。加强信息、网络等科技基础设施建设，形成企业、科研机构、高校紧密联系、共享共用的创新服务平台，促进科技资源高效配置和综合集成。发展技术和专利产权交易市场，加快科技成果向现实生产力转化。加强软科学研究工作。加大科普宣传力度，提高全民科技创新意识，增强产业技术创新能力，促进科技与经济发展的紧密结合。三是大力构建现代教育体系。继续优化重组教育资源，推进教育创新，放大优质教育资源效应。全面落实"以县为主"的农村义务教育管理体制，大力实施农村中小学布局调整，积极推进教育现代化工程，促进城乡教育和区域教育均衡发展。加快普及高中阶段教育，力争初中毕业生升学率达到85%。组建南通高等职教集团和中等职教集团，做大做强职业教育。构建全民教育体系，大力创建学习型城市。鼓励、引导和扶持社会力量举办非义务教育阶段学校，推进教育投资主体多元化，形成公办学校和民办学校共同发展的格局。加快南通大学园区建设，基本建成南通大学中心校区核心区。加强教师队伍建设，深入实施素质教育，确保教育质量领先地位。四是积极推进文化大市建设。把文化大市建设纳入国民经济和社会发展规划。以"五个一工程"为抓手，精心组织艺术生产，不断推出精品力作。加强"中国近代第一城"历史遗存的保护和修缮，抓紧实施博物苑新展馆建设，加快发展文博事业。加大基层文化设施建设投入，加强和改进基层文化工作。通过市场化运营、产业化整合，推动文化事业单位转型、文化产业升级，促进文化事业、文化产业与经济建设协调发展。加快发展广电事业，推广数字电视，提高有线电视覆盖率。全面启动省十六运办赛、参赛工作，广泛开展全民健身运动，发展体育产业，加快向体育强市迈进。五是切实提高公共卫生服务水平。强化政府公共卫生管理职能，完善疾病预防控制体系、突发公共卫生事件医疗救治体系、重大疫情信息网络体系和卫生执法监督体系，不断提高公共卫生服务质量，增强突发公共卫生事件应急处置能力。高度重视高致病性、传染性疫病防治工作，全面落实防治措施。加强农村卫生工作，改善农村卫生医疗条件，对贫困农民实行医疗救助。探索公立医疗机构经营权和所有权的分离，引导和支持社会资本参与重组或投资兴办医院。坚持计划生育基本国策，稳定低生育水平，提高出生人口素质，进一步完善计划生育特困家庭救助机制。维护未成年人、妇女、老年人、残疾人合法权益。积极发展地方志、档案、保密、气象、人防、无线电管理等事业，做好民族宗教工作。

　　（九）加强社会主义精神文明和民主法制建设，着力推动社会全面进步。坚持"三个文明"一起抓，为跨越发展提供不竭的精神动力和安定团结的良好环境。一是切实抓好思想道德建设。深入实施《公民道德建设实施纲要》，广泛开展社会公德、职业道德、家庭美德教育，大力倡导"爱国守法、明礼诚信、团结友善、勤俭自强、敬业奉献"基本道德规范，发扬光大精神文明"南通现象"，形成良好社会公共道德风尚，使崇尚创业成为新时代南通精神的重要内涵。深入开展群众性精神文明建设活动，整体推进文明村镇、文明社区、文明行业、文明单位等创建活动，倡导健康文明的生活方式，不断提高市民素质，提升城市文明程度，全力创建全国文明城市，争创全省江北第一文明城市群。支持驻通部队建设，再创全国"双拥模范城"。二是大力加强民主法制建设。认真执行人大及其常委会的决议、决定，自觉接受监督。积极支持人民政协工作，主动加强与各民主党派、工商联、无党派人士以及各人民团体的联系，广泛开展决策咨询，推进政府决策民主化、公开化和科学化。认真办好人大议案、代表建议和政协委员提案，不断提高办理质量和效率。进一步推进政务公开，坚持、完善政府新闻发言人制度，公示、听证制度和民主评议政风行风制度，扩大人民群众对事权、财权的知情、监督权。全面加快依法治市进程。继续大力实施依法治市"五年规划"，着力推进"四五"普法教育，增强国家公务员法治观念，提高广大人民群众法律素质。规范行政立法和行政执法行为，强化行政复议制度和行政执法监督。加强基层自治组织制度化、规范化建设，加快推进基层民主，市、县（市）区两级"民主法治示范村"、"民主法治示范社区"达到20%以上。建设"诚信南通"，整顿和规范市场经济秩序，着力建设公正、公平、公开的市场环境和社会诚信体系。三是全力维护社会政治稳定。完善具有鲜明南通特色的社会矛盾、纠纷大调解机制，高度重视做好信访工作。严格实行维护稳定工作责任制，妥善处置各类群体性事件。严格执行安全生产责任制。大力推进"平安南通"建设，强化社会治安综合治理，加强专职保安联防队伍建设，推行基层群防群治，着力构筑社会治安防控体系，严密防范、严厉打击各种刑事和经济犯罪。

　　（十）扎实做好扩大就业和社会保障工作，着力提高人民生活水平。关注民生，促进民富，继续把惠及人民群众的事情做好。一是把扩大就业放在经济社会发展更加突出的位置。坚持就业优先的原则和劳动者自主择业、市场调节就业和政府促进就业的方针，努力改善就业和创业环境，认真落实促进就业再就业的各项政策，加快城乡统一的劳动力市场建设步伐，建立适应市场经济发展的就业管理制度，进一步健全就业服务体系，促进劳动者充分就业。二是努力构筑与本地区经济发展水平相适应的较为完善的社会保障体系。继续巩固"两个确保"，完善"三条保障线"的衔接。全市养老、失业保险实行全覆盖。实行市区社会保险统筹，提高社会保障支撑能力。改革社会保险征缴方式，市区实行"五险合一"。促进社会保险扩面征缴，加快调整和完善有关配套政策，规范劳动关系，健全管理体制。建立新型农村合作医疗制度，稳步推进农村养老和最低生活保障。继续做好对口支援和第二批三峡移民安置工作。切实关注困难群体，加强社会救助体系建设，不断提高保障水平。三是全力改善城乡人民生活。提高城乡居民收入水平。鼓励劳动、知识、技术、管理和资本等生产要素按贡献参与分配，提高要素性、资产性、经营性收入的比重。千方百计增加城乡居民特别是中低收入居民的收入，稳步提高机关事业单位职工收入。切实关心离休干部和退休人员的生活，健全最低工资保障制度，建立企业职工和农民工收入维权机制，严禁拖欠职工工资。政府在日常工作中要坚持为人民群众多办实事、多办好事，同时本着与群众利益密切相关、受惠面广、迫切需要解决、年内可以完成的原则，今年还要突出重点，集中办好以下实事：（1）全年提供城镇就业岗位10万个，实现3.5万名城镇失业人员就业，净增就业人数3.3万人；农村劳动力转移新增6万人。（2）加速改善城乡居民饮水条件，西北片区域供水工程完成投资量1亿元，启动启海区域供水工程前期工作；新增农村改水受益人口不低于34万人。（3）实施"肉菜粮放心工程"，全市建立200万亩无公害农产品生产基地和60个以上无公害畜产品基地，全市大型农贸市场全面实行食品质量安全准入制度。（4）全面完成农村草危房改造任务；市区建设低价位普通商品房60万平方米，改造山芝庄居民住宅，消除最后一个"棚户区"。（5）优化小区环境，投入3300万元对市区新村出入口、积水地段、老旧住宅小区分期进行综合改造，提高道路、路灯等设施的完好率，实施市区1万户居民自来水一户一表工程，创造条件对老小区实施准物业管理。（6）整治市区骨干河道、水系，完成濠河周边污水截流工程，对濠河进行一次彻底疏浚；市区人均新增公共绿地1.2平方米。（7）完成农村公路改造2140公里，新建和改造桥梁361座，解决农村行路难问题；开工建设城闸大桥，畅通唐闸与主城区的连接。（8）大力发展城市公共交通，优化公交线路，市区新增公交车116辆，新辟线路14条，新增公交站台450个；70岁以上的老人免费乘坐市内公交车。（9）全市城镇职工基本医疗保险参保面达90%，新型农村合作医疗覆盖率达70%以上；加快城乡社区卫生服务站建设步伐，年内普及率达80%。（10）城市、农村低保标准均提高10%左右。继续扶持15000户贫困户，脱贫率确保60%，力争达到65%；发动社会各方面与5000名重点优抚对象结对帮扶，解决特殊困难。（11）农村"五保"老人集中供养率达到43%，年人均供养标准超过1650元；向下肢残疾者赠送轮椅1000辆。（12）实行110、119、122"三台合一"，进一步整合110社会联动资源，完善社会应急救援体系；全面设立社区警务室，保障群众安居乐业。

　　三、切实加强自身建设

　　面对跨越发展的新形势，我们必须认真贯彻党的十六大、十六届三中全会精神，按照市委的要求和人民的期望，围绕"创新、规范、高效、廉洁"的目标，实践"三个代表"，牢记"两个务必"，做到"为民、务实、清廉"。

　　（一）执政为民，建设服务型政府。树立正确的政绩观，权为民所用，情为民所系，利为民所谋，以服务发展为己任，以服务百姓为天职，把执政为民贯穿到政府工作的始终。围绕争创全省最佳办事环境的目标，进一步转变政府职能，继续深化行政审批制度改革，更好地服务经济、服务项目、服务投资主体、服务人民群众。继续大力削减行政审批事项，凡能通过市场竞争机制调节、行业组织和中介组织规范的自律性管理以及行政机关采用事后监督等方式予以规范的，均不再设定行政许可。创新行政审批方式，简化审批程序，优化审批环节，按照争创全省一流的要求，继续办好行政审批服务中心，坚持首问负责、并联审批、一站办结、一条龙服务，推行告知承诺制，提高行政服务效率。进一步巩固"三清"成果，切断产生"中梗阻"的利益链条，从机制上解决"中梗阻"的源头问题，使部门的权力与部门责任紧密挂钩，与部门利益彻底脱钩。建立完善为投资者排忧解难的重点大户挂牌保护制度、检查准入制度，在执法部门、窗口单位推行一次申告投诉查实下岗制，营造良好的政务环境。加快电子政务建设，推进政府管理创新，提高行政效能，降低行政成本。继续办好"市长热线电话"和"市长电子信箱"，完善市长接待日制度，更加全面深入地体察民情、了解民意，设身处地为群众着想，高度关注并采取有效措施，妥善处理新形势下的人民内部矛盾，解决好改制、征地、拆迁、环保等涉及群众切身利益的热点、难点问题，把好事办实，实事办好。

　　（二）求真务实，强化狠抓落实第一基本功。抓落实是真功，是硬功，也是政府工作的基本功。要大力弘扬求真务实精神，大兴求真务实之风，加强学习、开阔视野，解放思想、锐意创新，深入实际、勤于思考，尊重规律、力戒浮躁，以新标准、新招数、新干劲、新节奏狠抓落实，使追求的目标成为加快发展、为民谋利的实绩。高度重视做好基础性、全局性、应对性工作，强化统计等基础环节，有针对性地超前建立突发事件、重大问题应对工作预案，加强形势分析预测，善于研究解决经济社会发展的新情况、新问题。强化长远发展战略的研究，启动"十一五"规划的编制。强化重点工作推进机制，建立健全以工兴市、体制改革、招商引资、民营经济、江海开发、城市建设以及接轨上海等重要工作的组织推进体系，明确责任部门、责任人和完成时限，加强效能监察，确保重点工作推进有计划、有部署、有督查、有成效。对市人民代表大会确定的重点工作、重点项目和为民办实事项目，不折不扣地抓好落实。进一步完善目标管理机制，加大奖惩力度，实行政府部门主要负责人末位诫勉谈话制、领导干部引咎辞职制、责令辞职制，引导和激励广大行政人员脚踏实地，真抓实干，将更多的时间、精力集中到谋发展、抓落实上。

　　（三）依法行政，从严治政，切实加强廉政建设。要以《行政许可法》的实施为契机，进一步提高政府依法行政水平，逐步实现行政行为、行政程序的法定化，坚持通过法定方式和途径，按照法定权限和程序行使权力，履行职责。要认真落实党风廉政建设责任制，建立健全与社会主义市场经济体制相适应的教育、制度、监督并重的预防腐败体系，完善结构合理、配置科学、程序严密、制约有效的政府权力运作机制，严格执行"四大纪律八项要求"，促进领导干部廉洁自律。注重从源头上预防腐败，严格收支两条线管理，强化领导干部任期经济责任审计制度和机关内部审计监督制度，规范审批、理财和机关分配行为。进一步抓好纠正部门和行业不正之风、查处大案要案工作，依法重点查办领导干部违法违纪案件，特别是违反政治纪律的案件和贪污、受贿、挪用公款的案件。要始终保持谦虚谨慎、不骄不躁的作风，艰苦奋斗的作风，自觉接受法律监督、纪律监督、民主监督、舆论监督和群众监督，正确行使手中的权力，清正廉洁，乐于奉献，当好人民的公仆。

　　各位代表，当前我市正处于跨越发展的历史新阶段。让我们在省委、省政府和市委的领导下，以邓小平理论和"三个代表"重要思想为指导，全面贯彻落实党的十六大和十六届三中全会精神，以只争朝夕、奋发有为的精神状态，艰苦创业、追求卓越的坚定信念，脚踏实地、求真务实的工作作风，团结拼搏，开拓进取，全面推进我市改革开放和现代化建设的宏伟大业，为推进全市经济社会跨越发展、实现"两个率先"的目标而努力奋斗！
